# Global nutrition 1990–2015: A shrinking hungry, and expanding fat world

**DOI:** 10.1371/journal.pone.0194821

**Published:** 2018-03-27

**Authors:** Wen Peng, Elliot M. Berry

**Affiliations:** 1 Department of Public Health Nutrition, School of Medicine, Qinghai University, Xining, China; 2 Research Center, the Amity Foundation, Nanjing, China; 3 Department of Human Nutrition and Metabolism, Braun School of Public Health, Hebrew University Hadassah Medical School, Jerusalem, Israel; Institut de recherche pour le developpement, FRANCE

## Abstract

**Objectives:**

Following its publication in 2008, the Global Nutritional Index (GNI) which captures the triple burden of malnutrition, has been updated to assess the overall nutritional status and nutritional trends of countries, regions and the world, including both under-nutrition and over-nutrition.

**Methods:**

The GNI was modeled on the Human Development Index, using geometric means of three normalized indicators: protein-energy malnutrition (PEM, measured by Disability-Adjusted Life Years (DALYs) from PEM), micronutrient deficiency (MID, measured by DALYs from MID), and penalizing obesity (percent female obesity). GNI (range 0–1) was calculated from 1990–2015 for 186 countries, in seven World Bank income and WHO region groupings.

**Results:**

World GNI increased from 0.433 to 0.473 as decreased deficits overcompensated for the rise in obesity. GNI for African low- and middle-income countries (LMIC) (median 0.301 to 0.392) and South-East Asian LMIC (0.456 to 0.564) improved significantly (*P*<0.001), while for high-income countries (0.657 to 0.611) worsened significantly (*P*<0.001). GNI for American LMIC (0.459 to 0.457), European LMIC (0.571 to 0.575), Eastern Mediterranean LMIC (0.484 to 0.483) and Western Pacific LMIC (0.433 to 0.494) were unchanged. The disaggregation of the GNI showed that in nearly all the seven country groups there was a significant decrease in both PEM and MID (all *P*<0.01) (except in HIC where only PEM dropped), and a significant increase in obesity (all *P*<0.001).

**Conclusion:**

These trends are the result of the reciprocal changes between decreased under-nutrition and increased over-nutrition, which has become a major cause of malnutrition worldwide. We suggest, therefore, that future Sustainable Development Goals should include alongside “zero hunger”–“reduce obesity”.

## Introduction

The United Nations has declared 2016–2025 to be the Decade of Action on Nutrition. To enable prioritization for such Action, the state of world malnutrition and its trends requires monitoring information concerning the triple burden of malnutrition–undernourishment (too little energy intake), micronutrient deficiency (vitamin and mineral deficiencies, so-called “hidden hunger”) and obesity (excess energy intake). In 2015, it was estimated that 795 million people were undernourished globally [1], one-third to a half of the world population were affected by micronutrient deficiency (so-called “hidden hunger”) [2], while the number of adults with overweight/obesity in 2014 was more than 1.9 billion [3]. The Global Nutritional Index (GNI) was introduced in 2008 to capture this triple burden of malnutrition (both deficiency and excess), as it was the only index to penalize a country for obesity [4].

The GNI was modeled on the human development index (HDI) [5], using three indicators of nutritional status—undernourishment, micronutrient deficiency and over-nutrition [6], which affect populations worldwide. Following the GNI publication in 2008 [4], the current paper has updated the GNI calculation with a modified methodology, to show the time trends of global, regional and individual country’s nutritional status. In this paper, the GNIs’ trends and their disaggregation have demonstrated the overall nutritional status, and the main nutritional issues of individual countries, regions and the world, which are needed by nutritionists, public health professionals and policy makers alike.

## Materials and methods

### Indicators for the triple burden of malnutrition

Three updated indicators for the GNI have been used in this report: protein-energy malnutrition (PEM) (age-standardized Disability-Adjusted Life Years (DALY) rates per 100,000 due to PEM) [7], micronutrient deficiency (MID) (age-standardized DALY rates per 100,000 due to iron, vitamin A, iodine deficiency and other nutritional deficiencies such as zinc deficiency) [7,8] and excess (age-standardized prevalence of female obesity above 18 years old) [9]. Data for PEM and MID were from the Global Burden of Disease (GBD) Project [7], and for obesity, from WHO [9]. Since we did not have the combined prevalence of obesity for both females and males, and the values for females are usually higher than those for males, we decided to use the female obesity to calculate the GNI. The dataset used for the calculation was shown in S1 Dataset.

### Calculation of country-specific GNI

A unit-free score, using the maximum and minimum values of the three indicators was calculated for each country. The geometric mean of the (1 –dimension- specific- score) was utilized to calculate the GNI (range 0–1), which is to penalize high values of the three indicators, compared with arithmetic mean.

The basic formula for GNI has been updated as

$$GNI=\sqrt[{3}]{\left(1-P)\times (1-M)\times (1-E\right)}$$

Where P = relative score of protein-energy deficiency

            M = relative score of micronutrient deficiency

            E = relative score of nutritional excess

In which

$$P=\frac{lg(PEM)-lg(PEM\;min)}{lg(PEM\;max)-lg(PEM\;min)}$$

(PEM is the DALY rates lost from protein-energy deficiency)

$$M=\frac{lg(MID)-lg(MID\;min)}{lg(MID\;max)-lg(MID\;min)}$$

(MID is the DALY rates lost from micronutrient deficiency)

$$E=\frac{Excess-Excess\;min}{Excess\;max-Excess\;min}$$

(Excess is the prevalence of female obesity)

We conducted a log transformation for PEM and MID to normalize these scores. The maximum and minimum values for three indicators of the GNI from 1990 to 2015 are shown in Table 1. Prevalence of obesity was updated to 2014. Therefore, the values for 2014 were used for GNI2015 calculation.

**Table 1:** Maximum and minimum values for the indicators of the GNI from 1990–2015.

Indicator	Maximum (source)	Minimum (source)
**PEM**	7194.95 *	0.28
	Somalia (2010)	Singapore (2015)
**MID**	3285.49	126.30
	Somalia (1990)	Mauritius (2015)
**Excess**	54.5%	0.5%
	Samoa (2014)	Vietnam (1990)

*Two extreme outliers in North Korea in PEM in 1995 and 2000 (greater than 20,000) were excluded. The P score for these two extreme outliers were set as 1.

The maximum and minimum values were taken from the 186 countries listed for the years of 1990, 1995, 2000, 2005, 2010 and 2015.

An example of GNI calculation is shown in S1 File.

### Country grouping

Calculations were made for 186 countries classified into seven groups (n = 11–52) according to WHO and World Bank income categories, separating out high-income countries (HIC) from the six WHO regions for low- and middle-income countries (LMIC) [10–12]. The country grouping was shown in S1 Table. GNI and other index results are presented as median and *inter-quartile range (IQR)*.

### Statistical methods

Friedman Exact test was used for the GNI comparisons between different years, and the Holm-Bonferroni method was used to correct the post hoc pairwise multiple comparisons. Wilcoxon signed rank test was used for the PEM and MID comparisons between 1990 and 2015. Paired student t test was used to compare the prevalence of female obesity between 1990 and 2014.

## Results

### Time trends of global GNI and its indicators

**Fig 1:**
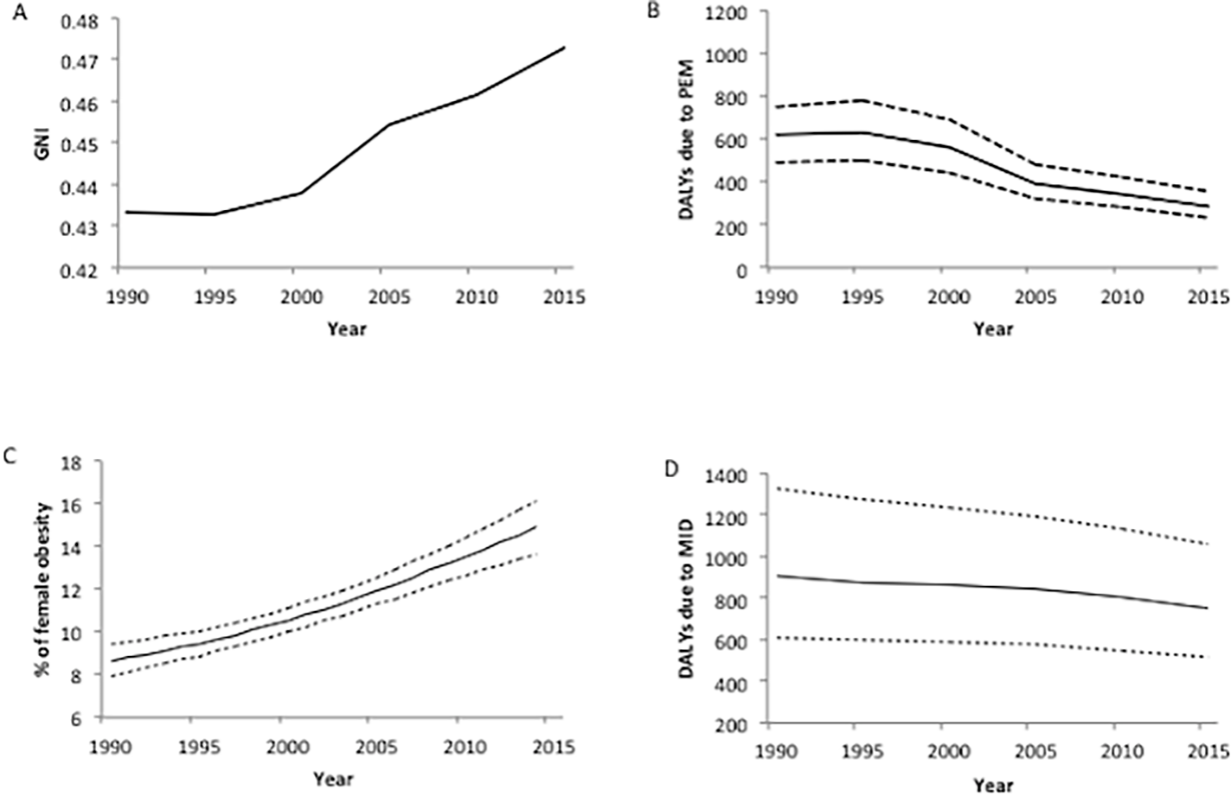
GNI and its indicators for the world from 1990 to 2015. PEM, protein-energy malnutrition. MID, micronutrient deficiency. Dotted lines above and below the lines in B, C and D represent the upper- and lower- limits of 95% uncertainty intervals.

Global collective GNI increased from 0.433 in 1990 to 0.473 in 2015 (Fig 1A). This improvement was mainly due to decreased PEM and MID (Fig 1B and 1D) offsetting the rise in obesity (Fig 1C)—PEM dropped more than 50% (619.5 *(498*.*2–784*.*4)* to 286.5 *(228*.*9–356*.*6)* DALYs); MID dropped approximately 20% (903.9 *(613*.*2–1329*.*2)* to 747.9 *(511*.*5–1060*.*8)* DALYs), while obesity increased by nearly 75% (8.6% *(7*.*9–9*.*4)* to 14.9% *(13*.*6–16*.*1*)).

### Time trends of regional GNI by WHO and World Bank groupings

**Fig 2:**
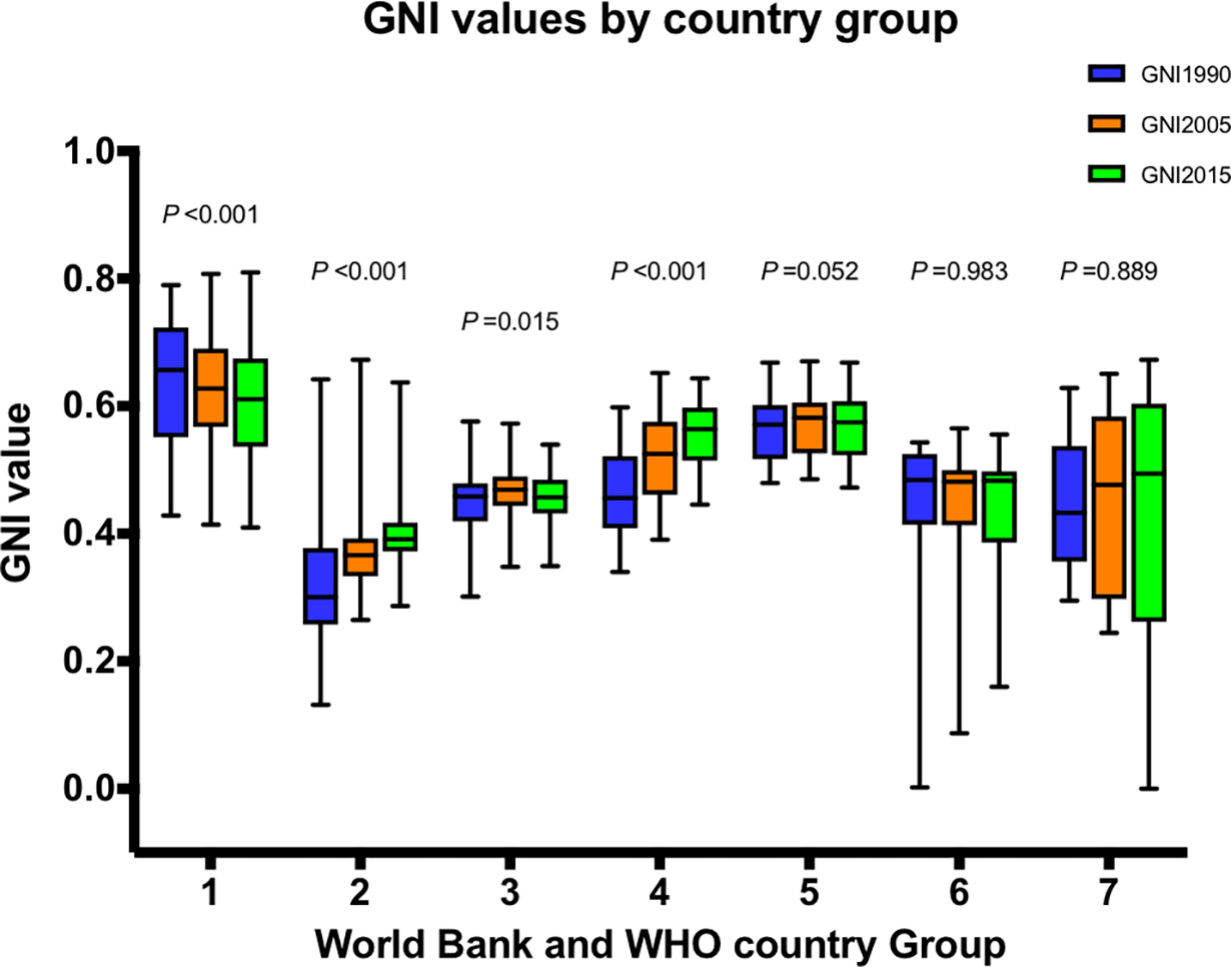
Box-whisker plots of GNI by World Bank and WHO group for 1990, 2005 and 2015. Groups—1, High-income countries (n = 52); 2, African low-and middle-income countries (LMIC) (n = 45); 3, American LMIC (n = 26); 4, South-East Asian LMIC (n = 11); 5, European LMIC (n = 20); 6, Eastern Mediterranean LMIC (n = 16); 7, Western Pacific LMIC (n = 16). Thick line in the box—median; upper- and lower- borders of box—75% and 25% percentile; whiskers—maximum and minimum. In group 6 and 7, because some country has one of the maximum values of the three indicators, the GNI of which appears to be zero when calculated by the geometric mean. P values are derived from Friedman’s Exact test.

Fig 2 demonstrates the different GNI time trends of the country groups. Although HIC (n = 52) remained the leading country group, their GNI worsened progressively from 0.657 in 1990 to 0.628 in 2005, to 0.611for 2015 (all *P* values *among and between years* <0.001). This counter-intuitive trend was caused by a dramatic– 50%–increase in obesity from 1990 to 2014 (from 16.8±7.1% to 25.3±8.8%, *P*<0.001). By contrast, the GNI for African LMIC (n = 45) improved significantly from 0.301 for 1990, to 0.366 for 2005, to 0.392 for 2015 (all *P* values *among and between years* <0.001), yet they remained the lowest ranking group. South-East Asian LMIC (n = 11) also improved from 0.456 for 1990 to 0.525 for 2005, and to 0.564 for 2015, (all *P* values *among and betwee*n *years* <0.05).

The GNI for American LMIC (n = 26) increased from 0.459 for 1990 to 0.469 for 2005 (*P value* = 0.027), then declined to 0.457 for 2015 (*P value* = 0.152), without overall improvement (*P value between 1990 and 2015 = 1)*, *(P value among years =* 0.015). European LMIC (n = 20) showed the same trend as American LMIC, from 0.571 for 1990 to 0.582 for 2005, to 0.575 for 2015, at borderline significance (*overall P* = 0.052).

Eastern Mediterranean LMIC (n = 16), 0.484 for 1990 to 0.483 for 2015 and Western Pacific LMIC (n = 16), 0.433 for 1990 to 0.494 for 2015, remained stable across the MDG timespan (both *P values across years* >0.800).

The GNI 1990, GNI 2005 and GNI 2015 for 186 individual countries are listed in S1 Table.

### Aggregation of top and bottom ranking countries for GNI

**Fig 3:**
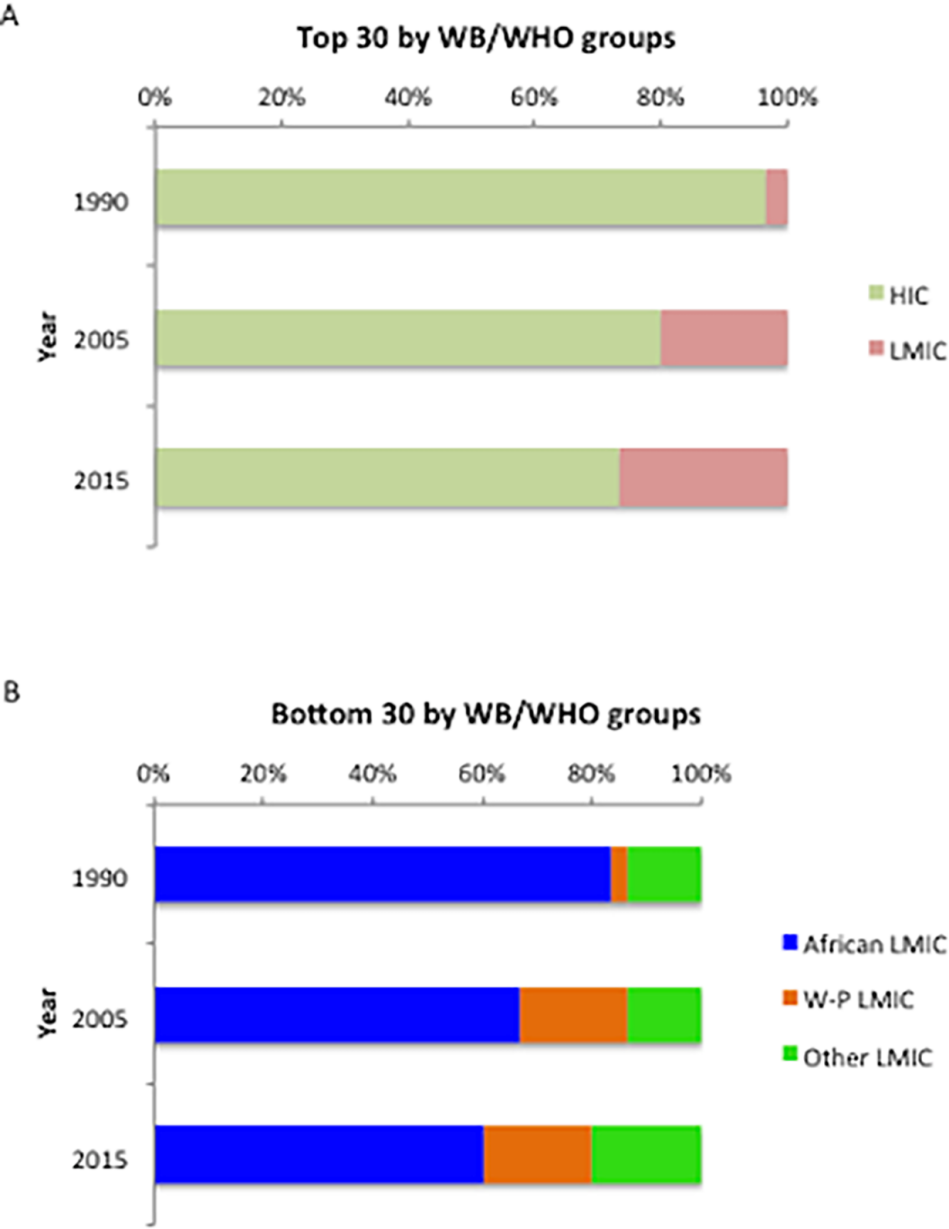
Top and bottom 30 countries in GNI ranking in 1990, 2005 and 2015. WB, World Bank; WHO, World Health Organization; HIC, high-income countries; LMIC, low- and middle-income countries; W-P, Western Pacific.

The deterioration in HIC, and the improvement in African LMIC, in nutritional status were also reflected in the top and bottom ranking countries for GNI. Among the top 30 countries, the percentage representation of HIC decreased progressively, over 25 years, from 96.7% in 1990, to 73.3% in 2015 (Fig 3A); among the bottom 30 countries, the number of African LMIC decreased steadily, from 83.3% in 1990, to 60.0% in 2015. In addition, six (20%) Western Pacific LMIC joined the bottom 30 from 2005 (Fig 3B).

Among the bottom 30 countries for GNI 2015, African LMIC and Western Pacific LMIC showed different patterns in the three dimensions of the GNI. In African LMIC, the median DALY rates due to PEM and MID were much higher than those in Western Pacific LMIC (PEM, 1169.2 *(692*.*1–1550*.*6)* vs. 190.0 *(146*.*4–469*.*2)*; MID 1290.0 *(1055*.*1–1408*.*8)* vs. 552.5 *(429*.*0–881*.*8))*, (both *P* <0.001), while the prevalence of female obesity was only one quarter that of Western Pacific LMIC (11.3±3.6% vs. 46.6±11.6%), (*P*<0.001). The different GNI patterns in bottom ranking countries showed that both under- and over-nutrition contributed to severe malnutrition.

### Disaggregation for GNI 1990 and GNI 2015 by WHO and World Bank groupings

**Fig 4:**
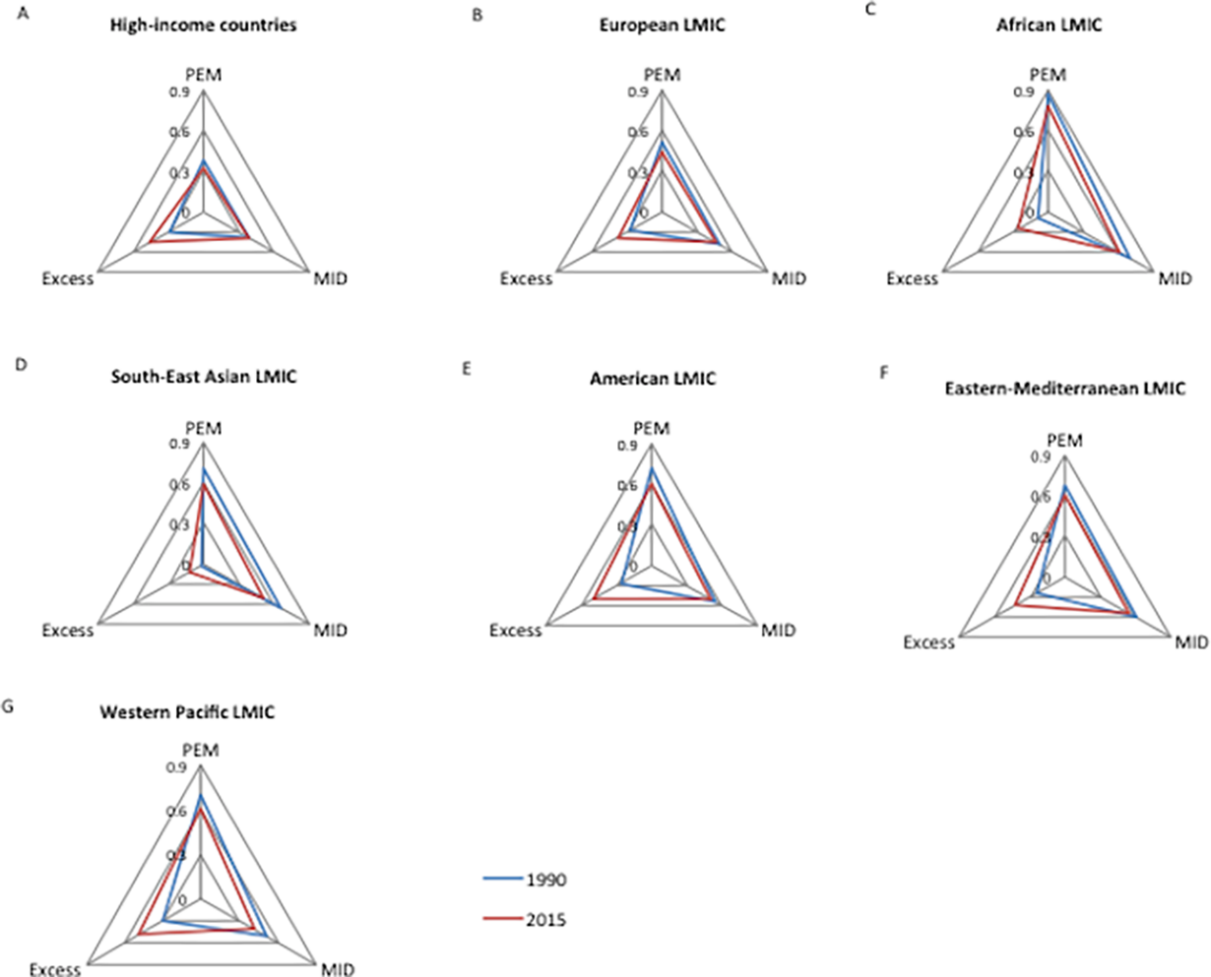
Disaggregation of GNI by country group for 1990 and 2015. LMIC, low- and middle- income countries; PEM, protein-energy malnutrition; MID, micronutrient deficiency. Each dimension of the triangle is represented by the mean of the dimension-specific score.

The GNI may be disaggregated to show the different trends of its three dimensions as in Fig 4. Zero indicates the lowest nutritional burden in a given dimension, while one represents the highest. Nearly all the seven country groups shared the same trends in the three indicators, with a significant decrease in both PEM and MID (all *P*<0.01) (except in HIC where only PEM dropped), and a significant increase in obesity (all *P*<0.001). Nevertheless, three patterns could be observed in the regional GNI.

The first was the pattern of HIC and European LMIC (Fig 4A and 4B). Both groups had relative low burdens of under-nutrition in 1990, with maximum mean scores for PEM and MID around 0.5. What differs between them is that the area of the triangle for European LMIC was larger than that for HIC and thus had a greater malnutrition burden with a similar pattern. Increased obesity was, in 2015, the direct cause for the worsening GNI for HIC who ranked, the third highest among the seven groups for nutritional excess (prevalence of female obesity 25.3±8.8%) after American LMIC (27.4±3.9%), followed by Western Pacific LMIC (26.8± 19.3%).

The second pattern was represented by African LMIC and South-East Asian LMIC (Fig 4C and 4D). In 1990, the dimension of excess in both groups was almost negligible (mean scores <0.01), while PEM and MID were major causes of malnutrition (mean scores >0.6). In 2015, excess increased dramatically, particular in African LMIC (prevalence of female obesity 5.3±4.7% to 14.4±7.4%), while the deficits shrank, yet remained the major causes of malnutrition. African LMIC had a larger triangular area than South-East Asian LMIC.

The third pattern was shown in the remaining three country groups (Fig 4E, 4F and 4G). In 1990, PEM and MID were the main causes of malnutrition, but by 2015 decreased deficits (both PEM and MID) and substantially increased excess have led to near equilateral triangles.

The three dimension–specific scores of GNI by country group for 1990 and 2015 are presented in S2 Table.

## Discussion

Substantial improvements in reducing hunger have been observed from 1990 to 2015. According to the FAO, sub-Sahara Africa is the region with the largest absolute improvement in PoU (Prevalence of Undernourishment) [1], and South Asia also achieved a sizeable reduction in nutritional deficits [1]. These trends have been reflected by the significant improvement in regional GNIs (Fig 2), and their disaggregation (Fig 4). The reduced hunger in both regions was also supported by the regional trends of global hunger index (GHI), though hunger was assessed using different indicators [13]. However, there are still challenges to reach the SDG-2 objective of zero hunger, particularly for countries/regions with political instability or frequent natural disasters [1]. Meanwhile, over-nutrition is becoming the major public health cause of malnutrition. The health and economic consequences of obesity are well-known including increased risk of cardiovascular disease, cancer, diabetes, osteoarthritis, chronic kidney disease, even directly associated with increased premature death [14]. In the bottom 30 GNI countries in 1990, nearly all were “hungry” countries (only one with a prevalence of female obesity >20%), while in 2015, ten out of 30 had a prevalence obesity greater than 20%.

It is also alarming that obesity prevalence has accelerated in developing countries, but seems to be attenuating in the developed world [15]. In the datasets we used for obesity, the top four groups with the *absolute increase* in prevalence of female obesity were all LMIC, and HIC ranked fifth among seven, leaving European LMIC and South-East Asian LMIC behind. These facts suggest that over-nutrition is overtaking undernutrition as the major cause of malnutrition. However, the attenuated increase in obesity in HIC may suggest that some progress is being made in combating this failure in public health action.

Some limitations to the GNI are noted. Firstly, the validity and calculation of the GNI relies on the quality of data which are mainly provided by individual countries and which may not be uniformly reliable. Secondly, it is an over-generalization to use a *single value* to represent the GNI for one country, particularly for large countries such as China, India or Brazil. In fact, we suggest that a subnational / regional GNI is more appropriate. Such an approach has been adopted in several countries for GBD 2013, and expanded further in GBD 2015 [16]. Thirdly, the current cut-off values for obesity (BMI 30kg/m^2^) may not be appropriate (too high) for Asian populations. However, it has been decided by WHO expert consultation, to maintain the universal WHO BMI cut-off values for classification, but the values for public health actions for Asians were lowered to 27.5 kg/m^2^ [17]. Lastly, the three components of GNI were inter-dependent, not only from a conceptual understanding, but also supported by their partial correlations. A positive association between PEM and MID (0.608), and a negative correlation between obesity and MID (-0.295) were observed when the third variable was adjusted in datasets for GNI-2015 (both *P values*<0.001). Rather surprisingly, there was no significant negative correlation between obesity and PEM (-0.018, *P* = 0.805). The data for GNI-1990 followed similar partial correlations to those for GNI-2015.

The Global Nutritional Index (GNI) can help international agencies and governments prioritize targets in combating malnutrition. As it is the only index to penalize a country for over-nutrition, the simple of calculation of the GNI and its easy disaggregation, can provide a practical tool to monitor and compare overall nutritional status within and between countries and groups. The GNI should be updated at regular intervals (as for the HDI) to facilitate policy making in linking food security and nutrition [18] and sustainability [19] to achieve the new Sustainable Development Goals, which should now be amended to include alongside zero hunger–reduce obesity.

## Supporting information

S1 File An example to calculate the GNI. (DOCX)

S1 Table The GNI for 186 countries for 1990, 2005 and 2015 according to WHO, World Bank groupings. (DOCX)

S2 Table Protein energy malnutrition, micronutrient deficiency and excess scores by country groups and year. (DOCX)

S1 Dataset The dataset used for GNI 1990–2015 calculation. (XLSX)

## References

[1] FAO, IFAD, WFP. The state of food insecurity in the world 2015 Meeting the 2015 international hunger targets: taking stock of uneven progress. FAO, Rome, 2015. 10.3945/an.115.009936 PMC4561839 27352453

[2] Miller DD, Welch RM. Food system strategies for preventing micronutrient malnutrition. Food Policy. 2013;42:115–128.

[3] WHO. Obesity and overweight Geneva: World Health Organization, 2016 Available from http://www.who.int/mediacentre/factsheets/fs311/en/(Cited Apr 6 2017).

[4] Rosenbloom JI, Kaluski DN, Berry EM. A global nutritional index. Food Nutr Bull. 2008;29:266–277. doi: 10.1177/156482650802900403 19227051 10.1177/156482650802900403

[5] UNDP. Human Development Index (HDI). New York: United Nations Development Program, 2016 Available from http://hdr.undp.org/en/content/human-development-index-hdi (Cited Aug 12 2017).

[6] Pinstrup-Andersen P. Agricultural research and policy for better health and nutrition in developing countries: a food systems approach. Agr Econ-Blackwell. 2007;37:187–198.

[7] Institute for Health Metrics and Evaluation (IHME). Global Burden of Disease Study 2015 (GBD 2015) Results Seattle, United States: IHME, 2016 2016. Available from http://ghdx.healthdata.org/gbd-results-tool (Cited Jul 10 2017).

[8] Black R. Micronutrient deficiency–an underlying cause of morbidity and mortality. Bull World Health Organ. 2003;81:79 12751414 PMC2572405

[9] WHO. Obesity (body mass index> = 30), age-standardized (%) estimates by country. Gevena: World Health Organization, 2017. Available from http://apps.who.int/gho/data/view.main.CTRY2450A?lang=en (Cited Apr 19 2017).

[10] WHO. Definition of region groupings Gevena: World Health Organization, 2017 Available from http://www.who.int/healthinfo/global_burden_disease/definition_regions/en/ (Cited Apr 19 2017).

[11] WHO. Alphabetical List of WHO Member States Gevena: World Health Organization, 2017 Available from http://www.who.int/choice/demography/by_country/en/ (Cited Apr 19 2017).

[12] World Bank. Data: World Bank Country and Lending Groups. Washington: World Bank, 2016 Available from https://datahelpdesk.worldbank.org/knowledgebase/articles/906519-world-bank-country-and-lending-groups. (Cited Apr 11 2017).

[13] International Food Policy Research Institue (IFPRI), Concern Worldwide, Welthungerhilfe. Global Hunger Index: getting to zero hunger Washington, DC/Dublin/Bonn: IFPRI, Concern Worldwide, Welthungerhilfe, 2016 Available from http://ebrary.ifpri.org/utils/getfile/collection/p15738coll2/id/130707/filename/130918.pdf (Cited Apr 19 2017).

[14] Lim SS, Vos T, Flaxman AD, Danaei G, Shibuya K, Adair-Rohani H, et al A comparative risk assessment of burden of disease and injury attributable to 67 risk factors and risk factor clusters in 21 regions, 1990–2010: a systematic analysis for the Global Burden of Disease Study 2010. Lancet. 2012;380:2224–2260. doi: 10.1016/S0140-6736(12)61766-8 23245609 10.1016/S0140-6736(12)61766-8 PMC4156511

[15] Ng M, Fleming T, Robinson M, Thomson B, Graetz N, Margono C, et al Global, regional, and national prevalence of overweight and obesity in children and adults during 1980–2013: a systematic analysis for the Global Burden of Disease Study 2013. Lancet. 2014;384:766–781. doi: 10.1016/S0140-6736(14)60460-8 24880830 10.1016/S0140-6736(14)60460-8 PMC4624264

[16] Editorial. GBD 2015: from big data to meaningful change. Lancet. 2016;388:1447 doi: 10.1016/S0140-6736(16)31790-1 27733277 10.1016/S0140-6736(16)31790-1

[17] WHO Expert Consultation. Appropriate body-mass index for Asian populations and its implications for policy and intervention strategies. Lancet. 2004;363:157–163. doi: 10.1016/S0140-6736(03)15268-3 14726171 10.1016/S0140-6736(03)15268-3

[18] Gould J. Nutrition: A world of insecurity. Nature. 2017;544:S6–S7. doi: 10.1038/544S6a 28445448 10.1038/544S6a

[19] Berry EM, Dernini S, Burlingame B, Meybeck A, Conforti P. Food Security and Sustainability: can one exist without the other? Public Health Nutr. 2015;16:1–10. 10.1017/S136898001500021X PMC10271846 25684016

