# Supplementary material for: Global nutrition 1990–2015: A shrinking hungry, and expanding fat world
Source: PLoS One. 2018 Mar 27;13(3):e0194821. doi: 10.1371/journal.pone.0194821 (PMC5870987; doi:10.1371/journal.pone.0194821)
Supplement: S1 File — (DOCX) [file pone.0194821.s001.docx]

**S1 Text An example to calculate the GNI**

As an example, we calculate the GNI2015 for Japan.

Its PEM is 11·65

Its MID is 450·61

Its Excess is 3·0

Therefore

P=$\frac{\lg\left(11\cdot65\right)-\lg\left(0\cdot28\right)}{\lg\left(7194\cdot95\right)-\lg\left(0\cdot28\right)}$= 0·367

M=$\frac{\lg\left(450\cdot61\right)-\lg\left(126\cdot30\right)}{\lg\left(3285\cdot49\right)-\lg\left(126\cdot30\right)}$= 0·390

E=$\frac{3\cdot0-0\cdot5}{54\cdot5-0\cdot5}$= 0·046

Japan’s overall GNI score== 0·717
