# Supplementary material for: Global nutrition 1990–2015: A shrinking hungry, and expanding fat world
Source: PLoS One. 2018 Mar 27;13(3):e0194821. doi: 10.1371/journal.pone.0194821 (PMC5870987; doi:10.1371/journal.pone.0194821)
Supplement: S1 Table — (DOCX) [file pone.0194821.s002.docx]

**S1 Table The GNI for 186 countries for 1990, 2005 and 2015 according to WHO, World Bank Groupings.**

| Country | Group | GNI-1990 | GNI-2005 | GNI-2015 | Rank 2015 |
| --- | --- | --- | --- | --- | --- |
| Singapore | 1 | 0·744 | 0·807 | 0·810 | 1 |
| South Korea | 1 | 0·685 | 0·742 | 0·743 | 2 |
| Austria | 1 | 0·778 | 0·748 | 0·730 | 3 |
| Japan | 1 | 0·756 | 0·715 | 0·717 | 4 |
| Finland | 1 | 0·730 | 0·718 | 0·708 | 5 |
| Greece | 1 | 0·721 | 0·714 | 0·707 | 6 |
| Italy | 1 | 0·790 | 0·723 | 0·703 | 7 |
| Netherlands | 1 | 0·739 | 0·714 | 0·703 | 7 |
| Iceland | 1 | 0·755 | 0·719 | 0·702 | 9 |
| Switzerland | 1 | 0·714 | 0·717 | 0·699 | 10 |
| Denmark | 1 | 0·709 | 0·677 | 0·679 | 11 |
| Sweden | 1 | 0·737 | 0·691 | 0·677 | 12 |
| Belgium | 1 | 0·754 | 0·688 | 0·676 | 13 |
| Vietnam | 7 | 0·540 | 0·598 | 0·673 | 14 |
| Cyprus | 1 | 0·725 | 0·683 | 0·671 | 15 |
| Norway | 1 | 0·768 | 0·712 | 0·670 | 16 |
| Luxembourg | 1 | 0·717 | 0·685 | 0·670 | 16 |
| Germany | 1 | 0·724 | 0·694 | 0·670 | 16 |
| Macedonia | 5 | 0·668 | 0·671 | 0·668 | 19 |
| Portugal | 1 | 0·681 | 0·675 | 0·660 | 20 |
| Brunei Darussalam | 1 | 0·686 | 0·671 | 0·658 | 21 |
| China | 7 | 0·575 | 0·639 | 0·657 | 22 |
| Malaysia | 7 | 0·628 | 0·651 | 0·656 | 23 |
| Andorra | 1 | 0·698 | 0·661 | 0·653 | 24 |
| Ireland | 1 | 0·732 | 0·674 | 0·646 | 25 |
| Thailand | 4 | 0·598 | 0·652 | 0·644 | 26 |
| Mauritius | 2 | 0·642 | 0·673 | 0·637 | 27 |
| United Kingdom | 1 | 0·721 | 0·667 | 0·632 | 28 |
| Ukraine | 5 | 0·577 | 0·625 | 0·631 | 29 |
| Serbia | 5 | 0·632 | 0·638 | 0·631 | 29 |
| New Zealand | 1 | 0·723 | 0·670 | 0·625 | 31 |
| Bosnia and Herzegovina | 5 | 0·605 | 0·614 | 0·621 | 32 |
| Mongolia | 7 | 0·582 | 0·622 | 0·615 | 33 |
| Poland | 1 | 0·641 | 0·621 | 0·615 | 33 |
| Malta | 1 | 0·667 | 0·632 | 0·614 | 35 |
| Spain | 1 | 0·659 | 0·631 | 0·611 | 36 |
| France | 1 | 0·645 | 0·624 | 0·611 | 36 |
| Hungary | 1 | 0·628 | 0·619 | 0·610 | 38 |
| Montenegro | 5 | 0·597 | 0·603 | 0·610 | 38 |
| North Korea | 4 | 0·538 | 0·575 | 0·608 | 40 |
| Australia | 1 | 0·690 | 0·632 | 0·603 | 41 |
| Bulgaria | 5 | 0·634 | 0·606 | 0·601 | 42 |
| Estonia | 1 | 0·557 | 0·595 | 0·599 | 43 |
| Slovakia | 1 | 0·621 | 0·610 | 0·599 | 43 |
| Sri Lanka | 4 | 0·522 | 0·581 | 0·598 | 45 |
| Israel | 1 | 0·654 | 0·616 | 0·598 | 45 |
| Myanmar | 4 | 0·498 | 0·548 | 0·597 | 47 |
| Croatia | 1 | 0·609 | 0·598 | 0·594 | 48 |
| Slovenia | 1 | 0·606 | 0·595 | 0·592 | 49 |
| Moldova | 5 | 0·566 | 0·581 | 0·592 | 49 |
| Romania | 5 | 0·603 | 0·588 | 0·585 | 51 |
| Belarus | 5 | 0·576 | 0·583 | 0·582 | 52 |
| Czech Republic | 1 | 0·605 | 0·581 | 0·577 | 53 |
| Nepal | 4 | 0·456 | 0·536 | 0·577 | 53 |
| Georgia | 5 | 0·586 | 0·605 | 0·576 | 55 |
| Armenia | 5 | 0·583 | 0·584 | 0·574 | 56 |
| Latvia | 1 | 0·549 | 0·576 | 0·573 | 57 |
| Canada | 1 | 0·641 | 0·608 | 0·572 | 58 |
| Philippines | 7 | 0·475 | 0·541 | 0·571 | 59 |
| Laos | 7 | 0·407 | 0·508 | 0·569 | 60 |
| Seychelles | 1 | 0·609 | 0·602 | 0·567 | 61 |
| Albania | 5 | 0·502 | 0·543 | 0·567 | 61 |
| Timor-Leste | 4 | 0·432 | 0·511 | 0·564 | 63 |
| Lithuania | 1 | 0·546 | 0·575 | 0·561 | 64 |
| Kyrgyzstan | 5 | 0·565 | 0·567 | 0·558 | 65 |
| Uzbekistan | 5 | 0·543 | 0·562 | 0·557 | 66 |
| Iran | 6 | 0·462 | 0·565 | 0·556 | 67 |
| Indonesia | 4 | 0·472 | 0·525 | 0·552 | 68 |
| Maldives | 4 | 0·445 | 0·512 | 0·550 | 69 |
| Turkmenistan | 5 | 0·542 | 0·547 | 0·543 | 70 |
| Costa Rica | 3 | 0·576 | 0·573 | 0·540 | 71 |
| Tunisia | 6 | 0·543 | 0·560 | 0·536 | 72 |
| Cambodia | 7 | 0·366 | 0·487 | 0·533 | 73 |
| United States | 1 | 0·647 | 0·565 | 0·528 | 74 |
| Yemen, Rep· | 6 | 0·528 | 0·521 | 0·525 | 75 |
| Colombia | 3 | 0·543 | 0·532 | 0·524 | 76 |
| Cuba | 3 | 0·543 | 0·551 | 0·523 | 77 |
| Chile | 1 | 0·620 | 0·556 | 0·523 | 77 |
| Tajikistan | 5 | 0·543 | 0·504 | 0·517 | 79 |
| Vanuatu | 7 | 0·531 | 0·520 | 0·515 | 80 |
| Bangladesh | 4 | 0·340 | 0·455 | 0·515 | 80 |
| Azerbaijan | 5 | 0·508 | 0·521 | 0·512 | 82 |
| Argentina | 3 | 0·512 | 0·513 | 0·500 | 83 |
| Russia | 5 | 0·501 | 0·500 | 0·498 | 84 |
| Morocco | 6 | 0·522 | 0·498 | 0·498 | 84 |
| Sudan | 6 | 0·465 | 0·466 | 0·496 | 86 |
| Uruguay | 1 | 0·524 | 0·515 | 0·495 | 87 |
| Syria | 6 | 0·504 | 0·493 | 0·492 | 88 |
| Brazil | 3 | 0·461 | 0·489 | 0·491 | 89 |
| Afghanistan | 6 | 0·411 | 0·457 | 0·490 | 90 |
| Iraq | 6 | 0·507 | 0·500 | 0·487 | 91 |
| Nicaragua | 3 | 0·462 | 0·479 | 0·485 | 92 |
| Cape Verde | 2 | 0·405 | 0·478 | 0·485 | 92 |
| Paraguay | 3 | 0·546 | 0·500 | 0·485 | 92 |
| Bhutan | 4 | 0·408 | 0·461 | 0·481 | 95 |
| Honduras | 3 | 0·466 | 0·479 | 0·480 | 96 |
| Libya | 6 | 0·533 | 0·497 | 0·479 | 97 |
| Mexico | 3 | 0·466 | 0·491 | 0·477 | 98 |
| Algeria | 2 | 0·464 | 0·478 | 0·476 | 99 |
| Kazakhstan | 5 | 0·491 | 0·485 | 0·474 | 100 |
| Solomon Islands | 7 | 0·459 | 0·467 | 0·473 | 101 |
| Peru | 3 | 0·419 | 0·469 | 0·473 | 101 |
| Turkey | 5 | 0·479 | 0·491 | 0·472 | 103 |
| Saudi Arabia | 1 | 0·535 | 0·507 | 0·468 | 104 |
| Oman | 1 | 0·450 | 0·476 | 0·468 | 104 |
| Pakistan | 6 | 0·424 | 0·454 | 0·467 | 106 |
| Lebanon | 6 | 0·507 | 0·491 | 0·466 | 107 |
| Dominican Republic | 3 | 0·420 | 0·469 | 0·466 | 107 |
| Ecuador | 3 | 0·419 | 0·444 | 0·465 | 109 |
| Ethiopia | 2 | 0·243 | 0·366 | 0·462 | 110 |
| Saint Lucia | 3 | 0·476 | 0·487 | 0·460 | 111 |
| Belize | 3 | 0·456 | 0·471 | 0·455 | 112 |
| Barbados | 1 | 0·488 | 0·475 | 0·450 | 113 |
| Dominica | 3 | 0·471 | 0·475 | 0·449 | 114 |
| Trinidad and Tobago | 1 | 0·496 | 0·489 | 0·446 | 115 |
| India | 4 | 0·354 | 0·391 | 0·446 | 115 |
| Bahrain | 1 | 0·481 | 0·453 | 0·445 | 117 |
| Grenada | 3 | 0·465 | 0·465 | 0·445 | 117 |
| Saint Vincent and the Grenadines | 3 | 0·431 | 0·448 | 0·444 | 119 |
| Panama | 3 | 0·488 | 0·452 | 0·440 | 120 |
| Jordan | 6 | 0·526 | 0·472 | 0·439 | 121 |
| Suriname | 3 | 0·418 | 0·441 | 0·437 | 122 |
| Togo | 2 | 0·373 | 0·410 | 0·435 | 123 |
| Sao Tome and Principe | 2 | 0·382 | 0·411 | 0·434 | 124 |
| The Bahamas | 1 | 0·458 | 0·452 | 0·432 | 125 |
| Bolivia | 3 | 0·411 | 0·441 | 0·432 | 125 |
| El Salvador | 3 | 0·454 | 0·446 | 0·431 | 127 |
| Fiji | 7 | 0·484 | 0·448 | 0·430 | 128 |
| Jamaica | 3 | 0·446 | 0·453 | 0·429 | 129 |
| Liberia | 2 | 0·301 | 0·397 | 0·429 | 129 |
| Cameroon | 2 | 0·383 | 0·417 | 0·428 | 131 |
| Uganda | 2 | 0·333 | 0·381 | 0·427 | 132 |
| Antigua and Barbuda | 1 | 0·462 | 0·453 | 0·426 | 133 |
| Kuwait | 1 | 0·485 | 0·460 | 0·422 | 134 |
| Gambia | 2 | 0·361 | 0·387 | 0·421 | 135 |
| Mozambique | 2 | 0·312 | 0·374 | 0·418 | 136 |
| Comoros | 2 | 0·279 | 0·380 | 0·417 | 137 |
| Swaziland | 2 | 0·440 | 0·399 | 0·415 | 138 |
| Namibia | 2 | 0·386 | 0·403 | 0·414 | 139 |
| United Arab Emirates | 1 | 0·428 | 0·414 | 0·410 | 140 |
| Qatar | 1 | 0·499 | 0·442 | 0·410 | 140 |
| Cote d'Ivoire | 2 | 0·349 | 0·399 | 0·409 | 142 |
| Equatorial Guinea | 2 | 0·270 | 0·385 | 0·405 | 143 |
| Rwanda | 2 | 0·228 | 0·341 | 0·405 | 143 |
| Ghana | 2 | 0·359 | 0·385 | 0·402 | 145 |
| Sierra Leone | 2 | 0·277 | 0·368 | 0·402 | 145 |
| Guinea | 2 | 0·280 | 0·371 | 0·400 | 147 |
| Botswana | 2 | 0·396 | 0·397 | 0·396 | 148 |
| Lesotho | 2 | 0·405 | 0·385 | 0·396 | 148 |
| Haiti | 3 | 0·318 | 0·377 | 0·395 | 150 |
| Zimbabwe | 2 | 0·430 | 0·389 | 0·392 | 151 |
| Guyana | 3 | 0·348 | 0·393 | 0·389 | 152 |
| Malawi | 2 | 0·267 | 0·344 | 0·387 | 153 |
| Angola | 2 | 0·232 | 0·343 | 0·385 | 154 |
| Burundi | 2 | 0·236 | 0·342 | 0·385 | 154 |
| South Africa | 2 | 0·387 | 0·383 | 0·384 | 156 |
| Benin | 2 | 0·286 | 0·359 | 0·382 | 157 |
| Guatemala | 3 | 0·301 | 0·367 | 0·381 | 158 |
| Gabon | 2 | 0·344 | 0·354 | 0·380 | 159 |
| Niger | 2 | 0·179 | 0·329 | 0·378 | 160 |
| Senegal | 2 | 0·292 | 0·342 | 0·378 | 160 |
| Congo | 2 | 0·319 | 0·351 | 0·377 | 162 |
| Papua New Guinea | 7 | 0·348 | 0·356 | 0·377 | 162 |
| Kenya | 2 | 0·336 | 0·359 | 0·377 | 162 |
| Zambia | 2 | 0·302 | 0·320 | 0·374 | 165 |
| Burkina Faso | 2 | 0·329 | 0·349 | 0·370 | 166 |
| Egypt | 6 | 0·433 | 0·400 | 0·369 | 167 |
| Mauritania | 2 | 0·269 | 0·338 | 0·369 | 167 |
| Nigeria | 2 | 0·254 | 0·327 | 0·358 | 169 |
| Tanzania | 2 | 0·250 | 0·322 | 0·358 | 169 |
| Guinea-Bissau | 2 | 0·295 | 0·327 | 0·352 | 171 |
| Venezuela | 3 | 0·451 | 0·348 | 0·349 | 172 |
| Congo, Dem. Rep. | 2 | 0·282 | 0·305 | 0·338 | 173 |
| Madagascar | 2 | 0·227 | 0·324 | 0·337 | 174 |
| Djibouti | 6 | 0·278 | 0·290 | 0·331 | 175 |
| Chad | 2 | 0·253 | 0·301 | 0·314 | 176 |
| Micronesia, Federated States of | 7 | 0·385 | 0·356 | 0·302 | 177 |
| Eritrea | 2 | 0·132 | 0·270 | 0·296 | 178 |
| Mali | 2 | 0·174 | 0·265 | 0·289 | 179 |
| Central African Republic | 2 | 0·262 | 0·290 | 0·287 | 180 |
| South Sudan | 6 | 0·183 | 0·264 | 0·268 | 181 |
| Kiribati | 7 | 0·313 | 0·268 | 0·249 | 182 |
| Marshall Islands | 7 | 0·295 | 0·248 | 0·208 | 183 |
| Somalia | 6 | 0·002 | 0·087 | 0·160 | 184 |
| Tonga | 7 | 0·353 | 0·245 | 0·090 | 185 |
| Samoa | 7 | 0·393 | 0·279 | 0·000 | 186 |

Groups–1, High-income countries; 2, African low-and middle-income countries (LMIC); 3, American LMIC; 4, South-East Asian LMIC; 5, European LMIC; 6, Eastern Mediterranean LMIC; 7, Western Pacific LMIC.

The GNI value of 0 means the country has the maximum value (or greater than the maximum value defined) in one of the three indicators from 1990-2015.
