# Supplementary material for: Global nutrition 1990–2015: A shrinking hungry, and expanding fat world
Source: PLoS One. 2018 Mar 27;13(3):e0194821. doi: 10.1371/journal.pone.0194821 (PMC5870987; doi:10.1371/journal.pone.0194821)
Supplement: S2 Table — (DOCX) [file pone.0194821.s003.docx]

**S2.1 Table Protein Energy Malnutrition scores by country groups and year.**

| Regions | | n | 1990 | |  | 2015 | | *P* |
| --- | --- | --- | --- | --- | --- | --- | --- | --- |
|  |  |  | mean | SD |  | mean | SD |  |
| HIC |  | 52 | 0·376 | 0·155 |  | 0·325 | 0·143 | <0·001 |
| LMIC | African Region | 45 | 0·868 | 0·069 |  | 0·773 | 0·071 | <0·001 |
|  | Region of the Americas | 26 | 0·716 | 0·089 |  | 0·608 | 0·075 | <0·001 |
|  | South-East Asian Region | 11 | 0·698 | 0·093 |  | 0·590 | 0·067 | <0·001 |
|  | European Region | 20 | 0·507 | 0·063 |  | 0·437 | 0·051 | <0·001 |
|  | Eastern Mediterranean Region | 16 | 0·670 | 0·148 |  | 0·596 | 0·148 | <0·001 |
|  | Western Pacific Region | 16 | 0·696 | 0·105 |  | 0·599 | 0·106 | <0·001 |

**S2.2 Table Micronutrient Deficiency scores by country group and year.**

| Regions | | n | 1990 | |  | 2015 | | *P* |
| --- | --- | --- | --- | --- | --- | --- | --- | --- |
|  |  |  | mean | SD |  | mean | SD |  |
| HIC |  | 52 | 0·384 | 0·083 |  | 0·385 | 0·068 | 0·796 |
| LMIC | African Region | 45 | 0·701 | 0·139 |  | 0·604 | 0·131 | <0·001 |
|  | Region of the Americas | 26 | 0·540 | 0·096 |  | 0·502 | 0·103 | 0·006 |
|  | South-East Asian Region | 11 | 0·656 | 0·092 |  | 0·511 | 0·103 | <0·001 |
|  | European Region | 20 | 0·487 | 0·101 |  | 0·465 | 0·089 | 0·012 |
|  | Eastern Mediterranean Region | 16 | 0·608 | 0·186 |  | 0·545 | 0·186 | <0·001 |
|  | Western Pacific Region | 16 | 0·512 | 0·162 |  | 0·423 | 0·147 | 0·001 |

**S2.3 Table Excess scores by country group and year.**

| Regions | | n | 1990 | |  | 2015 | | *P* |
| --- | --- | --- | --- | --- | --- | --- | --- | --- |
|  |  |  | mean | SD |  | mean | SD |  |
| HIC |  | 52 | 0·302 | 0·131 |  | 0·459 | 0·162 | <0·001 |
| LMIC | African Region | 45 | 0·089 | 0·088 |  | 0·257 | 0·137 | <0·001 |
|  | Region of the Americas | 26 | 0·260 | 0·059 |  | 0·499 | 0·071 | <0·001 |
|  | South-East Asian Region | 11 | 0·025 | 0·171 |  | 0·117 | 0·053 | <0·001 |
|  | European Region | 20 | 0·271 | 0·08 |  | 0·381 | 0·087 | <0·001 |
|  | Eastern Mediterranean Region | 16 | 0·247 | 0·176 |  | 0·426 | 0·229 | <0·001 |
|  | Western Pacific Region | 16 | 0·308 | 0·288 |  | 0·487 | 0·357 | <0·001 |
